# Beneficial role of healthy eating Index-2015 score & physical activity on COVID-19 outcomes

**DOI:** 10.1186/s40795-023-00727-8

**Published:** 2023-10-02

**Authors:** Mona A Hegazy, Marwa M Fouad, Samah Ahmed Abd elshafy, Dalia Abdelfatah, Rania M Lithy, Ahmed Abdelghani, Omar Ahmed Ashoush

**Affiliations:** 1 Internal medicine department, faculty of medicine, Cairo University, Cairo, 11562 Egypt; 2 Occupational and Environmental medicine department, Cairo University, Cairo, Egypt; 3 Nutrition and food Science department, Faculty of Home Economics, El-Azhar University, Cairo, Egypt; 4 Cancer Epidemiology and Biostatistics department, National Cancer Institute, Cairo University, Cairo, Egypt; 5 Endemic medicine department, faculty of medicine, Cairo University, Cairo, Egypt

**Keywords:** COVID-19, HEI-2015 score, Nutritional status, Global physical activity

## Abstract

**Background:**

Nutritional status and physical activity are essential to maintain a strong immune system. No definite pharmacological strategies for Coronavirus disease 2019 treatment are presently available, so natural enhancement of the immune system is in need. Our goal was to assess the correlation of healthy diet and physical activity on COVID-19 disease outcome.

**Methods:**

This cohort study was conducted on 68 adult patients who contracted mild (38) or moderate [30] cases of COVID-19, recruited via a convenience sampling technique from the outpatient clinic, Kasr Al-Aini Faculty of Medicine, Cairo University Hospital. Patients’ Healthy Eating Index (HEI) and degree of physical activity as measured by the Global Physical Activity Questionnaire were evaluated and linked with several inflammatory markers.

**Results:**

Most of patients with mild COVID-19 patients (92.1%) were physically active, compared to only 50% of moderate COVID-19. The total Metabolic Equivalent Task-min/week was positively correlated with the lymphocyte percentage. The median total HEI score was significantly higher in the patients with mild COVID-19 than with moderate COVID. Significant positive correlations observed among the lymphocyte count and total HEI-2015. There was approximately a 64% reduction in the probability of acquiring moderate COVID-19 illness for each unit rise in The HEI.

**Conclusion:**

Healthier nutrition and Physical activity correlated with reduced COVID-19 disease severity.

**Trial registration:**

The study was registered on clinical trial.gov maintained by the US National Library of Medicine (CinicalTrials.gov identifier = NCT04447144; https://clinicaltrials.gov/) (25/06/2020).

## Introduction

The severe acute respiratory syndrome coronavirus 2 (SARS-CoV-2)-caused coronavirus disease of 2019 (COVID-19) has become the most substantial worldwide health emergency since the 1918 influenza pandemic. On March 11, 2020, the World Health Organization (WHO) declared COVID-19 a global pandemic. Since then, the virus has continued to inflict waves of surging infections of high morbidity and mortality rates. Despite continued global mass vaccination efforts and the startlingly quick discovery of a COVID-19 vaccine, new variant strains pose a threat to undo the tremendous progress accomplished thus far in halting outbreaks [1].

The host’s immune system and SARS-CoV-2 interaction define how COVID-19 disease develops, and the immune response is determined by age, sex, nutritional status, physical status and genetics (HLA genes) [2]. Maintaining a healthy immune system against viral infections requires proper nutrition [3]. There is currently no proof that any supplement can improve immune function or treat or prevent viral infections. To combat COVID-19, we must be aware of the specific foods that can strengthen our immune systems [4]. A tool for assessing dietary quality, the Healthy Eating Index (HEI-2015), has been used to research the relationships between dietary quality and health outcomes [5].

The healthy eating index (HEI) is a measure for evaluating dietary quality, it has been used to examine associations between diet quality and health outcomes, such as risk for cardiovascular disease mortality [6].

Physical activity is considered one of the main gears of healthy living. In addition to prevention of excess body weight, systemic inflammation and chronic non-communicable diseases, a potential advantage with a postulated mechanism of physical exercise in reducing communicable diseases, including viral infections, is suggested [7].

Studies have shown that a variety of parameters, including the type, intensity, duration, and regularity of applied effort, might modulate the immunological response when exercising [8, 9]. In a report from US CDC included 25 studies provided consistent and conclusive evidence that physical activity reduced risk of severity of COVID-19 disease [10].

For viral illnesses like COVID-19, for which there are currently no pharmaceutical preventative or treatment options, nutritional strategies for enhancing immunity and regular physical exercise would be useful [11].

Therefore, the study aim was to assess the benefits of healthy diet and lifestyle on COVID-19 outcome by examining the correlations between WHO’s Global Physical Activity Questionnaire “GPAQ” and HEI-2015 with COVID-19 course and recovery in patients with mild and moderate disease.

## Patients and methods

### Study design and participants

Patients whose nasopharyngeal swab samples tested positive for COVID-19 by the polymerase chain reaction (PCR) between May 2021 and February 2022 were included in this longitudinal-cohort single-center investigation. They were gathered from the outpatient clinic of the Kasr Al-Aini Faculty of Medicine, Cairo University Hospital.

Adult patients aged 18–60 years had mild or moderate COVID-19 according to the WHO’s COVID- 19 criteria [12]. According to the Egyptian Ministry of Health and Population’s recommendations, patients with mild and moderate disease received paracetamol (1 g every 8 h), vitamin C (1 g), zinc (50 mg), acetylcysteine (600 mg), lactoferrin (2 sachets daily), prophylactic or therapeutic anticoagulant based on D-dimer level [13].

Patients with immunological illnesses, gastrointestinal conditions, or taking immunosuppressive drugs, those who were obese [body mass index (BMI) >30 kg/m^2^], those who were underweight (BMI < 18.5 kg/m^2^), patients with unintended weight loss for whom the reason remained under investigation, those on diet programs for weight loss, and those with severe COVID-19 infection according to WHO guidelines [12] were all excluded from the study.

Since there is no available data regarding HEI-2015 and GPAQ in patients with mild and moderate COVID-19, a pilot study was conducted with 24 patients: 12 mild and 12 moderate patients matched by age, sex, and BMI (normal and overweight up to 29.9 kg/m^2^). After comparing the patients with mild and moderate COVID-19 at a ratio 1:1, the average ± standard deviation (SD) HEI-2015 for patients with mild COVID-19 was 55.6 ± 2.8, whereas the average HEI-2015 in patients with moderate COVID-19 was 39.7 ± 9.3 (*p* < 0.001, highly significant), so the decision was made for the final sample size of 30 subjects per group (a total of 60 participants), we opted to examine 20 participants per group, compensated by 15% for the use of nonparametric tests, and compensated by 30% for suspected losses in order to be able to reject the null hypothesis, that the population mean HEI-2015 values of the patients with mild and moderate COVID-19 are equal with a probability (power) of 0.999. The test of this null hypothesis had a Type I error probability of 0.05. The PS program (https://vbiostatps.app.vumc.org/) was used to calculate the sample size.

The Research Ethics Committee, Faculty of Medicine, Cairo University (REC n-86-2020) accepted the study protocol, which complied with the Declaration of Helsinki 1975 [14] ‘s ethical principles. The data privacy policy was properly adhered to. Before enrolment, all participants gave their informed consent after receiving a thorough explanation of the study’s objective. An anonymous identifying code was used to preserve the patient’s privacy, and the electronic data was kept on a locked, password-protected computer. The study’s data were accessible to all authors, who also read and approved the final publication.

### Data collection

The participants’ age, sex, BMI, clinical symptoms, vital signs and underlying comorbidities (as hypertension, diabetes mellitus, chronic pulmonary, renal, hepatic or cardiac diseases) were obtained.

A nasopharyngeal swab was done 5 to 6 days after the first symptoms appeared. Swabs were quickly delivered to the lab in a viral transport medium. Real-time quantative reverse transcription-PCR (RT-qPCR) analysis using Taqman-based probes was performed for an in-vitro SARS-CoV-2 RNA transcription. Then, DNA amplification followed by fluorescence detection was performed. Reagents and materials used with the Rotor-Gene instrument (QIAGEN GmbH, Germany) for the analysis were obtained from VIASURE (Zaragoza, Spain).

The laboratory assessments consisted of blood count analysis, fasting blood sugar, kidney function tests, liver biochemistry tests (alanine aminotransferase, ALT; aspartate aminotransferase, AST; and total bilirubin), C-reactive Protein (CRP), D-dimer, ferritin, lactate dehydrogenase, and lipid profile analysis, which were performed within 24 h of the onset of symptoms after fasting for 12 h, and Chest computed tomography were performed at Faculty of Medicine, Cairo University Hospital, Cairo, Egypt.

### Dietary recalls and healthy eating index − 2015 (HEI-2015) calculation

The HEI-2015 measures 13 constituents that reflect the fundamental recommendations in the 2015–2020 Dietary Guidelines for Americans and includes two groupings: Adequacy components (nine food types we should eat enough in sufficient quantities) that represent the encouraged dietary elements, food groups and subgroups we need for overall good health (total fruit, whole fruit, total vegetables, greens and beans, whole grains, dairy products, total protein, seafood and plant protein, and fatty acids). Additionally, the HEI-2015 includes four moderation components that represent the dietary elements and food groups of recommended consumption limits (refined grains, sodium, added sugars, and saturated fat) [15]. Since obtaining an accurate estimate of long-term habitual food intake is crucial, according to literatures Food Frequency Questionnaire (FFQ) is the most used tool to assess individual usual dietary intake in nutritional epidemiological studies, especially for investigating the relationship between dietary and health outcomes [16].

The dietetic history of each participant was obtained from a local FFQ designed and applied by the National Nutrition Institute of Egypt for “evaluating the frequency of consumption of food daily, monthly, and yearly” to cover at least 1 year prior to COVID-19 infection. We assess FFQ over 1 year due to some Egyptian traditional and religious habits, as certain periods of the year we eat specific food, and some families are of low socioeconomic status that don’t eat meat except in the weekend, so they remember what they eat in some specific days. To reduce inter- and intra-observer bias, data collection team (two independent researchers) got three hours of training on the questionnaire, followed by a post-test to check their preparation and reporting quality.

Using the SPSS program version 26 (IBM SPSS Statistics for Windows, IBM Corp., Armonk, NY), the HEI-2015 score was determined using a simple HEI scoring algorithm technique in three steps: (1) Determine the set of foods being considered, (2) How much of each dietary component is present, and then (3) Calculate the ratio of dietary components to energy and assign a score. Higher scores indicate healthier diets, and a score of 100 indicates the ideal diet quality in accordance with the HEI-2015 dietary recommendations. The total score goes from 0 to 100 [17].

### Physical activity

To assess physical activity, we used the GPAQ version 2, which comprises 16 questions that assess 3 domains in which physical activity is performed: the work, travel to and from places, and recreational/leisure time activity. Sedentary behavior was also assessed. The domains of the questionnaire include questions regarding the performance of moderate-intensity and vigorous-intensity exercise continuously for ≥ 10 min a day for how many days a week and for how much time a day. The patient responses were calculated as Metabolic Equivalent Task (MET) minutes per week (METs-min/week), where the total time spent and energy expenditure in METs was calculated in all three domains of activity, with moderate activities consuming 4 METs, and vigorous activities consuming 8 METs. The Physical Activity Guidelines (2018) [18] adopted by the U.S. Department of Health and Human Services recommend that Adults need ≥ 150 min/week of moderate-intensity physical activity or 75 min of vigorous-intensity physical activity throughout the week for establishing and maintaining health benefits; or an equivalent combination of moderate- and vigorous-intensity activity accumulating ≥ 600 METs/min/week [19]. Physically active persons were those who achieved ≥ 600 METs-min/week, whereas inactive ones were those who achieved < 600 METs. High physically persons were defined as those who performed ≥ 3000 total METs-min/week [20].

### Data analysis

Data management and analysis were conducted using the SPSS program, version 26 (IBM SPSS Statistics for Windows, IBM Corp., Armonk, NY). The relevant means, standard deviations, medians, and/or ranges were used to summaries numerical data. Numbers and percentages were used to represent a categorical set of data. The frequency estimates were presented as percentages and figures. The normality of numerical data was examined using the Shapiro-Wilk test and the Kolmogrov-Smirnov test. Categorical data were compared between independent groups using the Chi square or Fisher’s tests, as appropriate. The Mann-Whitney test was used for numerical variables that weren’t normally distributed, whereas the Student’s t-test was used for comparisons between two groups. Spearman’s correlation coefficients were calculated to assess the strength of relationships between the non-normally distributed measures (*r*, range: -1 to + 1).

Factors with significance level < 0.10 were chosen to enter into stepwise logistic regression analysis in order to determine the independent effect of various factors on illness severity. The adjusted odds ratio (OR) and magnitudes of the effects of various covariates were determined through the use of logistic regression (disease severity). Additionally, the 95% Confidence Interval (95% CI), which was used to determine significance (any 95% CI that did not contain 1.0) was also calculated. Every test had a two-tailed design, and statistical significance was defined as p ≤ 0.05.

## Results

This study included 38 patients with mild and 30 moderate COVID-19. Females constituted 55.3% of the mild and 40% of the moderate patients. For the patients with mild and moderate COVID-19, age (mild: 37 ± 10 years; moderate: 41 ± 10 years; *p* = 0.066) and BMI (mild: 25.6 ± 3 Kg/m2; moderate: 26.7 ± 2.4 Kg/m2, *p* = 0.100) were matched. The duration of symptoms ranged from 2 to 41 days (median: 10 days, *p* = 0.011) in the patients with mild COVID-19, and ranged from 6 to 41 days (median: 15 days) in the patients with moderate COVID-19. There was no significant difference in the durations for conversion to negative PCR between the patients with mild and moderate COVID-19 (26 ± 10, 28 ± 6 days, respectively, *p* = 0.414).

Comparison of the comorbidities between the patients with mild and moderate COVD-19 were made for diabetes (7.9%, 20%, *p* = 0.169), hypertension (0%, 13.3%, *p* = 0.034), chronic lung disease (2.6%, 6.7%, *p* = 0.579), chronic liver disease (0%, 3.3% with non-applicable *p* value), and cardiac diseases (2.6%, 6.7%, *p* = 0.579), respectively. Comorbidities affected 13.2% of the patients with mild COVID-19 and 33.3% of the patients with moderate COVID-19 (*p* = 0.076).

Statistically significant lymphopenia (*p* = 0.043) and increased CRP (*p* = 0.001) were found in the moderate COVID-19 group (lymphocyte count of 1620/µl, CRP of 11.5 mg/L) relative to the mild COVID-19 group (lymphocyte count of 2080/µl, 2.4 mg/L), Table 1.

**Table 1:** Comparing mild and moderate COVID patients for physical activity according to GPAQ and different laboratory parameters

	Mild COVID patients (n = 38) Median (range)	Moderate COVID patients(n- 30) Median (range)	P value
PLT	238500 (125000–376000)	215000 (114000–503000)	0.327
TLC	6350 (2400–14600)	5400 (2600–13700)	0.361
**ANC**	3219 (400–8120)	2950 (600–9900)	0.747
**Neutrophil percent**	49.2 (0.4–81)	53.3 (0.7–83)	0.195
**Lymphocytic count**	2080 (35-4935)	1620 (37-4640)	0.043*
**Lymphocytes %**	35.6 (0.3–68)	34 (0.2–60.8)	0.486
** N/L ratio**	1.5 (0.2-5)	1.7 (0.3-8)	0.236
**Monocytes %**	5.1 (0-15.4)	3 (0-16.4)	0.542
**AST**	20 (10–45)	29 (15–60)	0.001*
**ALT**	22 (5–57)	26 (10–139)	0.067
**BIL-T**	0.1 (0-1.2)	0 (0-1.7)	0.745
**BIL-D**	0 (0-0.7)	0 (0-0.6)	0.895
**Creatinine**	0.8 (0.5–1.1)	0.9 (0-1.4)	0.203
**Urea**	26.5 (0–43)	24 (0-109)	0.228
**CRP**	2.4 (0.1–34)	11.5 (0-114)	0.001*
**Ferritin**	101 (0-486)	157.5 (0-867)	0.108
**LDH**	184.5 (0-380)	198.5 (0-545)	0.789
**d-Dimer( up to 0.44)**	0.2 (0-2.6)	0.2 (0–4)	0.649
	**Median (range)**	**Median (range)**	
**Moderate intensity activity in minutes/week**	10 (0–50)	5 (0–20)	0.051
**Physical activity (N, %)** **Inactive** **Active**	3 (7.9) 35 (92.1)	15 (50) 15 (50)	< 0.001*
**Physical activity** Low (< 600) Moderate (600–3000) High (≥ 3000)	3 (7.9) 28 (73.7) 7 (18.4)	15 (50) 15 (50) 0 (0)	< 0.001*
**Work MET min/week**	600 (0-6000)	150 (0-1200)	< 0.001*
**Travel to and from MET min/week**	600 (0-1200)	200 (0-600)	< 0.001*
**Sedentary behavior Hours/day**	5 (1–10)	6 (3-400)	0.014*
**Total MET min/week**	1200 (200–6600)	500 (0-1200)	< 0.001*

* P value < 0.05 is considered significant, PLT: Platelet, TLC: Total leucocytic count, ANC: Absolute neutrophilic count, N/L: Neutrophil lymphocyte ratio, AST: Aspartate aminotransferase, ALT: Alanine transaminase, BIL-T: Total bilirubin, BIL-D: Direct bilirubin, CRP: C-reactive protein, LDH: *lactate dehydrogenase*.

**Fig. 1:**
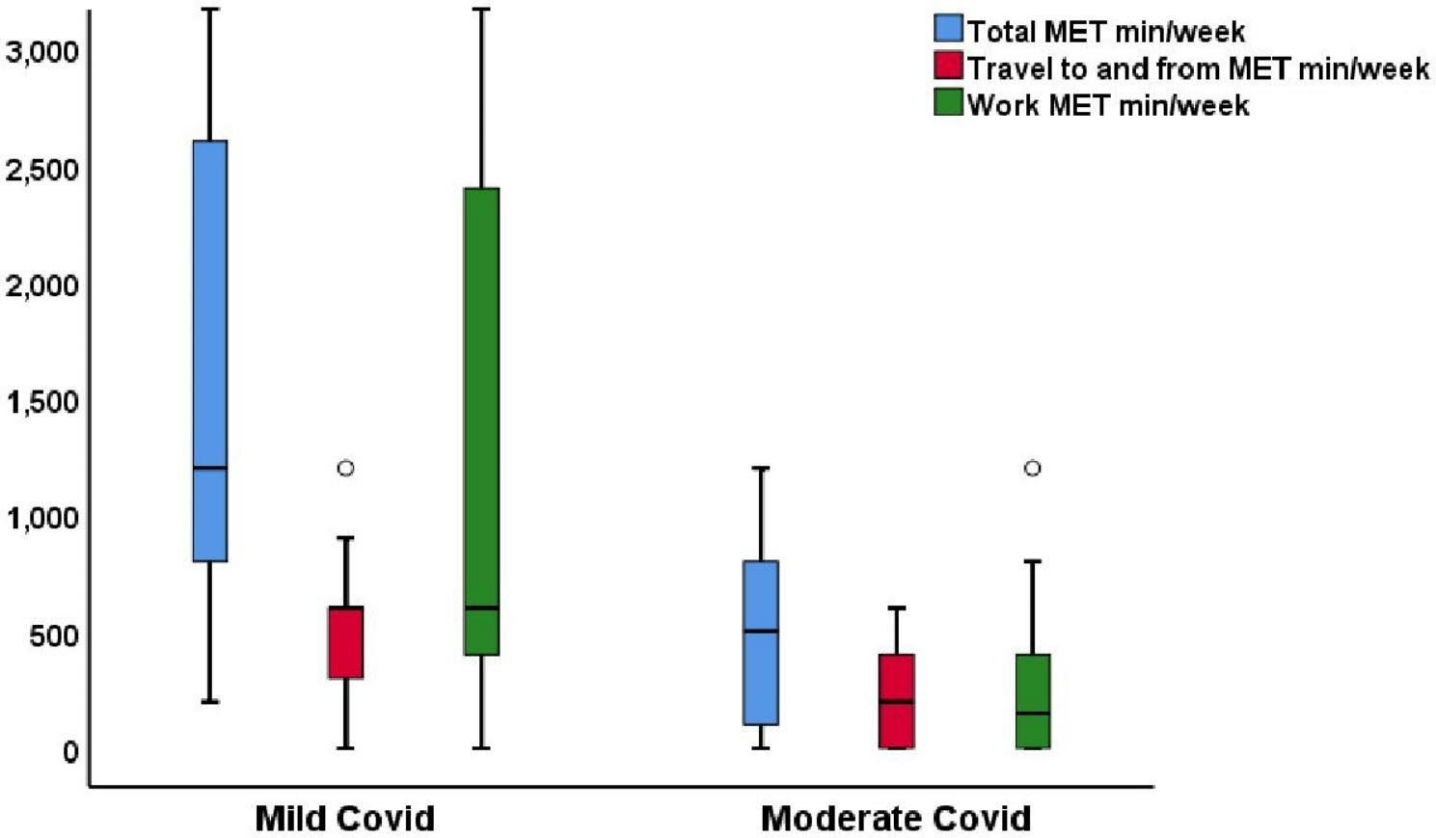
Box plot for GPAQ domains and total MET min/week for mild nd moderate COVID patients

A comparison showed that 92.1% of the mild COVID-19 group and 50% of the moderate COVID-19 group were physically active assessed by the GPAQ (*p* < 0.001). further classification of physical activity into low, moderate and high physical activity groups showed a significant difference (p < 0.001) between the mild and moderate COVID-19 groups, in which 73.7% of the patients with mild COVID-19 but only 50% of the patients with moderate COVID-19 engaged in moderate physical activity. Significant differences were found among the domains of GPAP-assessed physical activity (*p* < 0.001) and travel to and from places (*p* < 0.001), whereas none of the patients in the third domain of recreational or leisure activity had a history of regular physical activity. The total METs-min/ week for moderate-intensity activity (none of the studied groups had any vigorous-intensity activity) was significantly higher in the mild COVID-19 group than in the moderate COVID-19 group (*p* < 0.001), whereas sedentary behavior was significantly higher in the moderate COVID-19 groups than in the mild COVID-19 (*p* = 0.014) (Table 1) (Fig. 1).

Correlation analysis of physical activity with inflammatory markers revealed that; there were no significant correlations between the total METs-min/week, moderate-intensity activity, and any of the inflammatory markers in the mild COVID-19 patients, whereas there was a significant moderate-to-good positive correlation between the total METs-min/week and lymphocyte percentage(*r* = 0.53, *p* = 0.003). In addition to a significant fair negative correlation between the total METs-min/week and neutrophil lymphocyte ratio (*r* = -0.47, *p* = 0.009) in the moderate COVID-19 patients (Table 2).

**Table 2:** Correlation between total MET min/week, moderate intensity activity, and inflammatory markers, age and duration to negative PCR conversion among mild and moderate COVID patients

	Mild COVID patients (n = 38)				Moderate COVID patients (n = 30)					
	Total MET min/week		Moderate intensity activity (min/week)		Total MET min/week		Moderate intensity activity (min/week)			
	R	P value	r	P value	r	P value	r	P value		
**Age (Years)**	-0.30	0.069	-0.03	0.856	-0.23	0.221	0.11	0.509		
**Duration to negative PCR**	---	---	---	---	---	---	-0.24	0.204		
**HEI**	-0.05	0.764	0.20	0.239	0.18	0.337	---	---		
**Lymphocytic count**	-0.06	0.723	0.30	0.069	0.35	0.059	0.18	0.290		
**Lymphocytic (%)**	0.07	0.677	0.13	0.423	0.53	0.003**	0.08	0.616		
** N/R**	-0.02	0.903	-0.25	0.126	-0.47	0.009***	-0.16	0.348		
**CRP**	-0.18	0.286	0.07	0.674	-0.35	0.062	0.24	0.151		
**Ferritin**	-0.07	0.644	-0.01	0.967	-0.30	0.110	0.32	0.047*		
**LDH**	-0.18	0.291	-0.12	0.477	-0.10	0.585	-0.08	0.649		
**d-Dimer**	0.01	0.996	-0.05	0.790	-0.27	0.155	0.26	0.113		

N/L: Neutrophil lymphocyte ratio, r is the correlation coefficient & it ranges from − 1 to + 1, p value < 0.05 is considered significant.

*Significant fair positive correlation.

** Significant moderate to good positive correlation.

*** Significant fair negative correlation.

Assessment by HEI-2015 of diet quality in the studied population revealed a significantly higher HEI-2015 in the mild COVID-19 group [median: 56.5 (range: 50–71)] than in the moderate group [median: 41.5 (range: 23–52)], (*p* = 0.001) (Table 3). Saturated fat, refined grains, and sugar, which are three of the four moderation components in the HEI-2015 dietary components that should be limited or consumed in small amounts, were consumed in significantly higher amounts in the moderate COVID-19 group.

**Table 3:** Comparing mild and moderate COVID patients according to HEI-2015

	Covid severity		P value
	Mild COVID patients (n = 38)	Moderate COVID patients (n = 30)	
	Median (range)	Median (range)	
**Total fruit**	1.1 (0-2.8)	0.4 (0-2.7)	0.103
**Whole fruit**	2.5 (0-5.8)	1.2 (0–5)	0.116
**Total vegetable**	0.8 (0-2.3)	0.4 (0-2.8)	0.040*
**Green & beans**	3 (0-7.8)	2.3 (0-6.2)	0.034*
**Whole grains**	5.8 (1.8–10)	5.8 (0–10)	0.880
**Dairy**	0.8 (0-5.3)	0.8 (0-4.8)	0.890
**Total protein**	5 (1.4-5)	5 (4.2-5)	0.537
**Sea food& plant protein**	3.4 (0–5)	2.6 (0–5)	0.127
**Fatty acids**	0	0	1.000
**Refined grains**	8 (3–10)	4 (0–8)	< 0.001*
**Sodium**	9 (2–10)	8 (2–10)	0.142
**Add sugar**	10 (4–10)	6 (0–10)	0.001*
**Saturated fat**	8 (5–10)	2.5 (0–9)	< 0.001*
**HEI**	56.5 (50–71)	41.5 (23–52)	< 0.001*

*P value < 0.05 is considered significant.

**Fig. 2:**
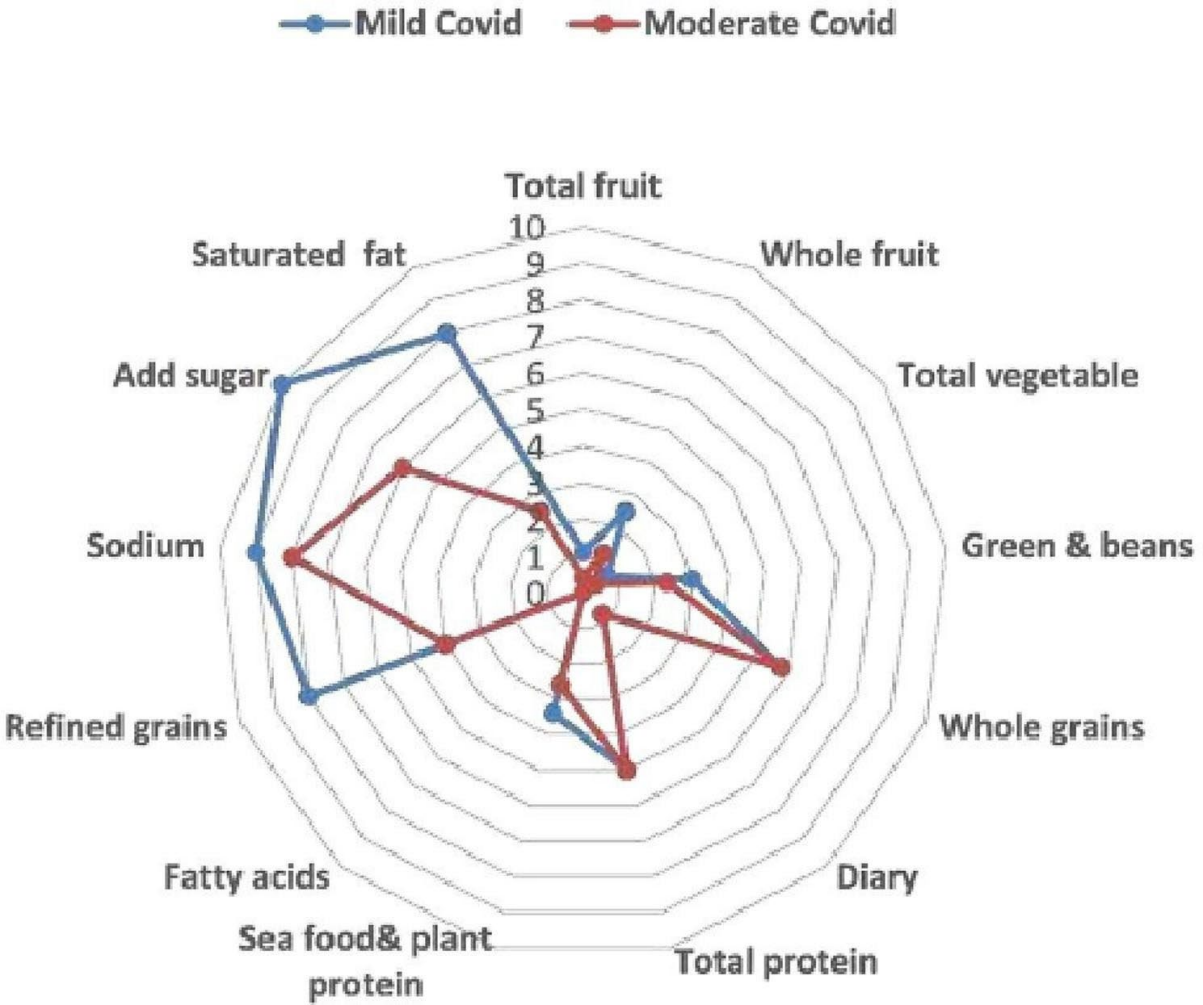
Radar plot for the 13 dietary components of HEI-2015 among mild and moderate COVID patients

Assessment of the nine food components that should be consumed in sufficient amounts to provide the nutrients needed for good health, there was a significantly higher ingestion of the total vegetables, greens, and beans in the mild COVID-19 group. Consumption of dairy products, whole grains, total protein, and fatty acids was similar between the mild and moderate COVID-19 groups (Fig. 2) (Table 3).

A significant fair positive correlation was observed among all COVID-19 patients between the lymphocytic count and HEI-2015 scores (*r* = 0.33, *p* = 0.005) and added sugar (r: 0.27, p 0.025). There was a significantly fair positive correlation present between added sugar and LDH level in the moderate COVID-19 group (*r* = 0.39, *p* = 0.032) (Table 4).

**Table 4:** Correlation between HEI-2015 total score, added sugar and inflammatory markers among COVID patients

	Whole COVID patients (n = 68)				Mild COVID patients (n = 38)				Moderate COVID patients (n = 30)			
	HEI		Added sugar		HEI		Added sugar		HEI		Added sugar	
	r	P value	R	P value	r	P value	r	P value	r	P value	r	P value
**Age (Years)**	-0.09	0.460	--	--	0.11	0.509	---	---	0.27	0.146	--	--
**Duration of symptoms**	-0.12	0.333	--	--	--	--	---	---	---	---	--	--
**Duration to negative PCR**	--	--	--	--	-0.24	0.204	---	---	-0.17	0.638	--	--
**HEI**	--	--	--	--	--	--	---	---	---	---	--	--
**Lymphocytic count**	0.33	0.005*	0.27	0.025*	0.18	0.290	0.26	0.123	0.31	0.100	0.18	0.335
**Lymphocytic (%)**	--	--	0.11	0.383	0.08	0.616	0.21	0.202	0.12	0.516	-0.10	0.642
** N/R**	--	--	-0.10	0.506	-0.16	0.348	-0.22	0.194	-0.12	0.537	-0.19	0.307
**CRP**	-0.22	0.071	-0.11	0.384	0.24	0.151	-0.10	0.594	0.30	0.108	0.29	0.118
**Ferritin**	-0.03	0.810	0.10	0.892	0.32	0.047*	0.10	0.791	0.16	0.415	0.12	0.513
**LDH**	0.002	0.988	0.10	0.496	-0.08	0.649	-0.10	0.562	0.29	0.125	0.39	0.032*
**d-Dimer**	0.08	0.512	0.14	0.266	0.26	0.113	0.26	0.122	0.15	0.426	-0.10	0.968

N/L: Neutrophil lymphocyte ratio, r is the correlation coefficient & it ranges from − 1 to + 1, p value < 0.05 is considered significant.

*Significant fair positive correlation.

** Significant moderate to good positive correlation.

*** Significant fair negative correlation.

Multivariate analysis was performed to measure the independent effects of all factors that affect the severity of COVID-19 disease, and factors with a significance level of *p* < 0.100 were selected to enter into stepwise logistic regression. The variables included in the model were: age, BMI, comorbidities, exercise and HEI-2015 score. The analysis showed that HEI-2015 was the only significant factor (*p* = 0.006), odds ratio = 0.36, 95% CI = 0.18–0.74), standard error = 0.37) and regression coefficient = -1.02). The regression coefficient showed the effect of each variable after controlling for the effects of the other variables in the model. For every unit increase in HEI-2015 score, there was about a 64% reduction in the risk of having moderate COVID-19.

## Discussion

Starting in late 2019 to early 2020, the novel SARS-CoV-2 virus rapidly spread from country to country and caused the worldwide COVID-19 pandemic that has continued into 2022. Most patients are asymptomatic or have mild disease. However, a considerable percentage of people have extensive pneumonia that can advance to hypoxemic respiratory failure, shock, multiple organ dysfunctions, and death [21]. COVID-19 vaccinations are thought to be extremely important to prevent and control COVID-19 because immunization is one of the health strategies that works the best and most affordably to avoid infectious diseases, Case series of vaccine-breakthrough infections also have been reported by variants of concern “VOCs,” such as the delta variant [22].

To our knowledge no previous study has correlated HEI-2015 with infectious diseases. High scores on the HEI-2015 suggest a healthy eating pattern that is high in fruits and vegetables, whole grains, dietary fiber, lean proteins, and unsaturated fatty acids and low in refined grains, sodium, added sugars, and saturated fats. Our study aim was to assess the effects of healthy diet and physical activity on COVID-19 disease outcomes examining correlation among the HEI-2015 and physical activity GPAQ with the disease course and recovery in mild- and moderate COVID-19 patients.

The main findings in our study were as follows: the median total HEI score was higher in the patients with mild COVID-19 than in those with moderate COVID-19. There was a significantly positive correlation among all COVID-19 patients between the lymphocyte count and HEI-2015 (*p* = 0.005). The GPAQ results showed that, 92.1% of the patients with mild COVID-19 patients were physically active (≥ 600 METs-min/week), compared to 50% of moderate COVID-19. The total METs-min/week was significantly and positively correlated with the lymphocytic percentage (*p* = 0.003) and negatively correlated with the neutrophil lymphocyte ratio (, *p* = 0.009) in patients with moderate COVID-19. The HEI-2015 was the main factor affecting the severity of COVID-19 disease, as shown by the finding that for every unit increase in the HEI-2015 score, there was about a 64% reduction in the risk of having moderate COVID-19.

A prior study on influenza patients showed that a good diet decreased the patients’ hospitalization rate, which is evidence that a healthy food pattern likely reduces the risk of severe COVID-19 [23]. On the other hand, alterations in some inflammatory markers caused by a bad diet may result in systemic inflammation [24].

A properly balanced diet, rich in all macronutrients and micronutrients, affects the functioning of the immune system positively. Enriched foods or a balanced diet including vitamins and minerals C, A, E, zinc, and vitamin D contribute to fighting and preventing the proliferation of SARS-CoV-2 virus cell particles and minimizing mortality and complications resulting from COVID-19 disease. The relationships described are important in that they also have their role in post- COVID prophylaxis and affect the building of lifelong immune reserves protecting against repeat infections and the development of a dangerous course of infection [25].

Our study assessed dietary habits 1 year prior to infection instead of what the patients recalled eating over the most recent 24-hours period because anorexia, fever, and gastrointestinal disturbance during infection can change the quality and quantity of the food choices by the patients. Besides, ensuring that the healthy lifestyle is maintained for an individual over one year gives a clue that micro and macro nutrients needed for proper immunity are provided. Analysis of the FFQ revealed that; saturated fat, refined grains, and sugar were consumed in significantly higher amounts by patients with moderate COVID-19 than those with mild disease. The patients with mild COVID-19 were found to have significantly higher ingestion of the total vegetables, greens, and beans than those with moderate COVID-19, and they had non-significantly higher consumption of total fruits, whole fruits, and seafood and plant protein. Consumption of dairy products, fatty acids, whole grains, and total protein were more or less similar between the two COVID-19 groups.

A previously published study that assessed the nutritional habits of COVID-19 patients found that individuals who consumed less fruit and poultry in their regular diets had more severe forms of the illness, and patients who drank more black tea appeared to have a higher chance of developing severe illness [26]. Our study findings agree with those of a previous study on respiratory viral infection involving influenza patients, where inactivity and low fruit and vegetable diet were linked to a higher likelihood of influenza hospitalization [23].

The present study results regarding the individual items of added sugar, total vegetables, greens and beans within the HEI- 2015 score, are in agreement with our previously published work on 200 COVID-19 patients who were evaluated using our novel ESSAP score (Exercise, Sugar consumption, Sleeping hours, Antibiotics, and Prebiotics consumption), which revealed that daily consumption of prebiotic-containing foods and sugar consumption of less than two teaspoons resulted in a milder disease and quick virus clearance [27].

A significantly fair positive correlation was observed among all COVID-19 patients between the lymphocyte count and HEI-2015 (*p* = 0.005). The total HEI-2015 score was positively correlated with serum ferritin among the patients with mild COVID-19 (*p* = 0.047).

Good dietary quality as measured by the HEI-2015 was associated with lower CRP and interleukin (IL)-6 concentrations, as well as white blood cell counts and its constituents. Significant relationships were also found for CRP, neutrophils, lymphocytes, and their neutrophil: lymphocyte ratio with total fruit intake in a previous study that examined the relationships between the HEI-2015 score and a range of inflammatory biomarkers in a cross-sectional sample of 1989 men and women There were no connections discovered between dairy, total protein meals, refined carbohydrates, or sodium and any inflammatory biomarkers [28].

Most of our mild patients where physically active (92.1%), and 73.7% of them showed moderate physical activity compared with only 50% of the patients with moderate COVID-19 than in the patients with mild COVID-19 (*p* < 0.001), whereas sedentary behavior was significantly higher in the patients with moderate COVID-19 than in the patients with mild COVID-19 (*p* = 0.014).

Physical activity has been investigated and reported to have a negative impact on severe COVID-19 results. The odds of hospitalization were 2.26 times higher for COVID-19 patients who continuously undertook sedentary behavior during the two years prior to the pandemic (OR 2.26; 95% CI: 1.81–2.83) than for COVID-19 patients who consistently met physical activity recommendations. Additionally, those who were consistently inactive had higher risks of being admitted to an intensive care unit (OR 1.73, 95% CI 1.18–2.55) and dying (OR 2.49, 95% CI 1.33–4.67) [29]. Even low-to-moderate intensity exercise decreased the likelihood of acquiring severe COVID-19 symptoms, which are linked to increased mortality [30].

Physical inactivity is typically linked to increased BMI and a higher likelihood of developing diabetes, both of which are comorbidities linked to negative COVID-19 results [31]. However, it has been discovered that a sedentary lifestyle increases mortality of COVID-19 hospitalized patients regardless of other risk variables (hazard ratio 5.91 (1.80-19.41); p = 0.003) [32].

A previous study involving 206 COVID-19 patients found that: patients with lower METs min/week not only developed a more severe form of the disease, but also demonstrated a statistically significant relationship between the duration of signs and symptoms and level of moderate to vigorous physical activity [26].

Our study revealed a significant moderate-to-good positive correlation between total METs-min/week and lymphocytic percentage (*r* = 0.53, *p* = 0.003) in addition to a significantly fair negative correlation between total METs-min/week and the neutrophil: lymphocyte ratio (*r* = -0.47, *p* = 0.009) among patients with moderate COVID-19.

The immune system is greatly changed by regular exercise. While prolonged or high-intensity exercise without adequate rest might cause lower cellular immunity, which can increase the propensity for infectious diseases, moderate-intensity exercise boosts cellular immunity [33]. Epidemiologic research have consistently found lower levels of white blood cell counts, CRP, IL-6, IL-18, tumour necrosis factor-alpha, and other inflammatory biomarkers in individuals who engaged in more physical activity and fitness [34, 35].

Finally, it appears that the effects of nutritional habits and physical activity are crucial factors related to COVID-19 disease severity. During the COVID-19 pandemic it is important to stay active and to exercise regularly in addition to maintaining healthy dietary habits, not only as a way to maintain good health, but especially to increase immunoprotective and immunoregulatory activity.

Nevertheless, there are limitations to our study, the number of patients was small to examine the combination/interaction of HE and PA in the explanation of COVID-19 outcomes, there is some sort of recall bias and subjectivity in FFQs as some patients may exaggerate or even underestimate what they eat for social and psychological factors, there was an unequal distribution in the disease severity groups, and there were no patients with severe COVID-19. Future studies in a greater number of patients with a wider range of COVID-19 severity may provide results that are more broadly applicable.

### Conclusions

Healthy eating habits and Physical activity might correlate with COVID-19 disease severity and the inflammatory markers.

### Recommendations

Individuals should perform a regular variable intensity exercise on a daily basis and strive for a high HEI-2015 by consuming healthy nutrition to help improve immune responses, which will provide greater protection against exposure to the SARS-COV-2 virus and reduce the risk of developing severe COVID-19.

## Data Availability

All data of the study may be made available upon reasonable request from the corresponding authors.

## References

[1] Aleem A, Akbar Samad AB, Slenker AK. Emerging variants of SARS-CoV-2 and novel Therapeutics against Coronavirus (COVID-19). In: StatPearls [Internet]. Treasure Island (FL): StatPearls Publishing; updated 2021Jun 2; 2021 Jan-. Available from: https://www.ncbi.nlm.nih.gov/books/NBK570580/.

[2] Li X, Geng M, Peng Y, Meng L, Lu S (2020). Molecular immune pathogenesis and diagnosis of COVID-19. J Pharm Anal.

[3] Aslam MF, Majeed S, Aslam S, Irfan JA (2017). Vitamin S:key role players in boosting up immune response, a mini review. Vitam Min.

[4] Haug A, Brand-Miller JC, Christophersen OA, McArthur J, Fayet F, Truswell S (2007). A food “lifeboat”:food and nutrition considerations in the event of a pandemic or other catastrophe. Med J Aust.

[5] Nutrition and your health. : 2015–2020 Dietary guidelines for Americans, 8th edition Washington, DC: US Government Printing Office; 2015.

[6] Onvani S, Haghighatdoost F, Surkan PJ, Larijani B, Azadbakht L (2017). Adherence to the healthy eating Index and Alternative Healthy Eating Index dietary patterns and mortality from all causes, cardiovascular disease and cancer: a meta-analysis of observational studies. J Hum Nutr Diet.

[7] Suzuki K, Hekmatikar AH, Jalalian S, Abbasi S, Ahmadi E, Kazemi A, Ruhee RT, Khoramipour K. The Potential of Exerkines in Women’s COVID-19: A New Idea for a Better and More Accurate Understanding of the Mechanisms behind Physical Exercise. Int. J. Environ. Res. Public Health. 2022 Nov 24;19(23):15645. 10.3390/ijerph192315645 PMC9737724 36497720

[8] Laddu DR, Lavie CJ, Phillips SA, Arena R (2021). Physical activity for immunity protection: inoculating populations with healthy living medicine in preparation for the next pandemic. Prog Cardiovasc Dis.

[9] Simpson RJ, Katsanis E (2020). The immunological case for staying active during the COVID-19 pandemic. Brain Behav Immun.

[10] https://www.cdc.gov/coronavirus/2019-ncov/downloads/clinical-care/E-Physical-Inactivity-Review.pdf.

[11] Khoramipour K, Basereh A, Hekmatikar AA, Castell L, Ruhee RT, Suzuki K. Physical activity and nutrition guidelines to help with the fight against COVID-19. J Sports Sci. 2021 Jan 2;39(1):101-7. 10.1080/02640414.2020.1807089 32842905

[12] World Health Organization. Clinical management of COVID-19: interim guidance, May 27 2020. World Health Organization. ; 2020. Available at: https://www.who.int/publications/i/item/clinicalmanagement-of-covid-19.

[13] Egypt Ministry of Health and Population. Diagnosis and treatment protocol for COVID 19.Cairo: Egypt Ministry of Health and Population.2020. Available at: http://www.mohp.gov.eg/JobsDetails.aspx?job_id=3061.

[14] World Medical Association Inc (2009). Declaration of Helsinki. Ethical principles for medical research involving human subjects. J Indian Med Assoc.

[15] Reedy J, Lerman JL, Krebs-Smith SM, Kirkpatrick SI, Pannucci TE, Wilson MM, Subar AF, Kahle LL, Tooze JA (2018). Evaluation of the healthy eating Index-2015. J Acad Nutr Diet.

[16] Shim JS, Oh K, Kim HC (2014). Dietary assessment methods in epidemiologic studies. Epidemiol Health.

[17] Krebs-Smith SM, Pannucci TE, Subar AF, Kirkpatrick SI, Lerman JL, Tooze JA, Wilson MM, Reedy J (2018). Update of the healthy eating Index-2015. J Acad Nutr Diet.

[18] US Department of Health and Human Services. Physical activity guidelines for Americans, 2nd edition. 2018. Washington, DC: US Department of Health and Human Services; 2018. Available at https://www.health.gov/PAGuidelines/Lastupdated.

[19] WHO. GPAQ: global physical activity questionnaire, 2.0 version. Department of Chronic Diseases and Health Promotion, WHO 2010. (Available at:http://www.who.int/chp/steps/resources/GPAQ_Analysis_Guide.pdf.

[20] WHO. The WHO STEPwise approach to noncommunicable disease risk factor surveillance: WHO STEPS surveillance manual. WHO., 2017. [Available at:. (Available at https://www.who.int/ncds/surveillance/steps/manual/en/.

[21] Veiga VC, Prats JAGG, Farias DLC (2021). Effect of tocilizumab on clinical outcomes at 15 days in patients with severe or critical coronavirus disease 2019: randomised controlled trial. BMJ.

[22] Kustin T, Harel N, Finkel U, Perchik S, Harari S, Tahor M, Caspi I, Levy R, Leshchinsky M, Dror SK, Bergerzon G. Evidence for increased breakthrough rates of SARS-CoV-2 variants of concern in BNT162b2-mRNA-vaccinated individuals. Nat Med. 2021 Jun;14:27:1. 10.1038/s41591-021-01413-7 PMC8363499 34127854

[23] Charland KM, Buckeridge DL, Hoen AG, Berry JG, Elixhauser A, Melton F, Brownstein JS. (2013) Relationship between community prevalence of obesity and associated behavioral factors and community rates of influenza-related hospitalizations in the United States. Influ Other Respir Viruses 2013; 7:718–728. 10.1111/irv.12019 PMC5781204 23136926

[24] Casas R, Sacanella E, Estruch R (2014). The immune protective effect of the Mediterranean diet against chronic low-grade inflammatory diseases. Endocr Metab immune Disord drug targets (formerly current drug targets-immune. Endocr Metab Disord).

[25] Iddir M, Brito A, Dingeo G, Del Fernandez SS, Samouda H, La Frano MR, Bohn T (2020). Strengthening the Immune System and reducing inflammation and oxidative stress through Diet and Nutrition: considerations during the COVID-19 Crisis. Nutrients.

[26] Tavakol Z, Ghannadi S, Tabesh MR, Halabchi F, Noormohammadpour P, Akbarpour S, Alizadeh Z, Nezhad MH, Reyhan SK. Relationship between physical activity, healthy lifestyle and COVID-19 disease severity; a cross-sectional study. J Public Health (Berl) 2021 Feb 4:1–9. 10.1007/s10389-020-01468-9 PMC7858040 33558839

[27] Hegazy MA, Ashoush OA, Hegazy MT, Wahba M, Lithy RM, Abdel-Hamid HM, Abdelfatah D, Ibrahim MH, Abdelghani A. Beyond probiotic legend: ESSAP gut microbiota health score to delineate COVID-19 severity. Br J Nutr 2021 Jun 7:1–34. 10.1017/S0007114521001926 PMC8410748 34096487

[28] Millar SR, Navarro P, Harrington JM, Perry IJ, Phillips CM. Dietary quality determined by the healthy eating Index-2015 and biomarkers of chronic low-grade inflammation: a cross-sectional analysis in middle-to-older aged adults. Nutrients. 2021 Jan;13:222. 10.3390/nu13010222 PMC7828829 33466696

[29] Després JP. Severe COVID-19 outcomes—the role of physical activity. Nat Rev Endocrinol 2021 Jun 10; 17:451–2. 10.1038/s41574-021-00521-1 PMC8191438 34112985

[30] Zadow EK, Wundersitz DWT, Hughes DL, Adams MJ, Kingsley MIC, Blacklock HA, Wu SSX, Benson AC, Dutheil F (2020). Gordon BACoronavirus (COVID-19), Coagulation, and Exercise: interactions that May Influence Health Outcomes. Semin Thromb Hemost.

[31] Sallis R, Young DR, Tartof SY, Sallis JF, Sall J, Li Q, Smith GN, Cohen DA. Physical inactivity is associated with a higher risk for severe COVID-19 outcomes: a study in 48 440 adult patients. Br J Sports Med. 2021 Apr 8;36. 10.1136/bjsports-2021-104080 33849909

[32] Salgado-Aranda R, Pérez-Castellano N, Núñez-Gil I, Orozco AJ, Torres-Esquivel N, Flores-Soler J, Chamaisse-Akari A, Mclnerney A, Vergara-Uzcategui C, Wang L, González-Ferrer JJ. Influence of baseline physical activity as a modifying factor on COVID-19 mortality: a single-center, retrospective study. Infect Dis Ther. 2021 Jun;10(2):801–14. 10.1007/s40121-021-00418-6 PMC7955903 33715099

[33] Pedersen BK, Hoffman-Goetz L (2000). Exercise and the immune system: regulation integration and adaption. Physiol Rev.

[34] Parsons TJ, Sartini C, Welsh P, Sattar N, Ash S, Lennon LT, Wannamethee SG, Lee IM, Whincup PH, Jefferis BJ (2017). Physical activity, sedentary behavior, and inflammatory and hemostatic markers in men. Med Sci Sports Exerc.

[35] Wedell-Neergaard AS, Krogh-Madsen R, Petersen GL, Hansen ÃM, Pedersen BK, Lund R, Bruunsgaard H (2018). Cardiorespiratory fitness and the metabolic syndrome: roles of inflammation and abdominal obesity. PLoS ONE.

